# Physical Activity, Resilience, Sense of Coherence and Coping in People with Multiple Sclerosis in the Situation Derived from COVID-19

**DOI:** 10.3390/ijerph17218202

**Published:** 2020-11-06

**Authors:** María Mercedes Reguera-García, Cristina Liébana-Presa, Lorena Álvarez-Barrio, Lisa Alves Gomes, Elena Fernández-Martínez

**Affiliations:** 1 SALBIS Research Group, Faculty of Health Sciences, Campus of Ponferrada, University of León, 24401 Ponferrada, Spain; mercedes.reguera@unileon.es (M.M.R.-G.); elena.fernandez@unileon.es (E.F.-M.); 2 Department of Nursing and Physiotherapy, Faculty of Health Sciences, Campus of Ponferrada, University of León, 24401 Ponferrada, Spain; lalvb@unileon.es; 3 Nursing School, Minho University, 4710-057 Braga, Portugal; lgomes@ese.uminho.pt

**Keywords:** multiple sclerosis, COVID-19, physical activity, resilience, sense of coherence, coping

## Abstract

The confinement forced by COVID-19 can have repercussions on the health of people diagnosed with multiple sclerosis. The objective of this study is to analyze the relationships between physical activity, a sense of coherence, resilience and coping among people diagnosed with Multiple Sclerosis during the health emergency situation. To achieve this goal, this transversal descriptive study included 84 patients that belonged to multiple sclerosis associations during the period of confinement. Participants filled out the Physical Activity (IPAQ-SF), Sense of Coherence (SOC-13), Resilience Scale (ER-14) and coping (COPE-28) questionnaires. The results showed that the average age was 46.9 and that 67.9% had Relapsing Remittent Multiple Sclerosis diagnosed on average 13.9 years ago. They had a high degree (33.3%) and moderate degree (34.5%) of physical activity, high levels of resilience, while the level of a sense of coherence was average and the most commonly used strategies for coping were active confrontation and religion. Physical activity was not related to the rest of the studied variables, but there were correlations between the other variables. The people with multiple sclerosis who belong to patient associations have remained physically active during the obligatory confinement period and have elevated degrees of resilience and an average sense of coherence, as well as using suitable coping strategies, which is why the social-health resource of belonging to a patient association could be boosting these variables that are beneficial to their health.

## 1. Introduction

The world is currently going through a pandemic due to a new strain of coronavirus, the severe acute respiratory syndrome (SARS-CoV-2). There are many aspects that remain unknown about this illness. At the moment there are no treatment strategies, nor is there a vaccine [1], and many countries have adopted social distancing and/or quarantining to break the chain of transmission of the COVID-19 illness. In Spain, a state of alarm was declared in order manage the health crisis situation brought about by COVID-19 in March 2020, the Royal Decree 463/2020 of 14 March [2], in response to the growing number of cases of coronavirus in Spain; it imposed a national quarantine as a health measure. On 21 June 2020, the country entered the dubbed “new normality”, introducing health measures such as the use of facemasks and social distancing. Current research [3] referring to the psychological impact of quarantine suggests that it is necessary to take into account the stress factors resulting from the situation (duration, fear of infection, frustration, fear, boredom, anger, insufficient information, lack of basic supplies, financial losses and stigma) in order to ensure that the experience of quarantining be as tolerable as possible for the population [3,4].

It is not known how duration might affect the different collectives of people who already suffer from prior illnesses such as multiple sclerosis (MS). MS is chronic and autoimmune, affecting the central nervous system [5] and with an unstable clinical course with a wide range of cognitive and neurological effects [6]. It affects women more than men (3:1) [7] and is usually diagnosed between 20 and 40 years of age. Certain characteristics of MS, such as its unknown origin, its unpredictable course, its multiple symptoms and the current nonexistence of a definitive cure, affect the adherence to the treatment as well as the quality of life [8]. MS patients suffer functional deterioration, fatigue, psychological anxiety and limitations in their social relationships, all of which leads to a lesser quality of life for these people at both the physical and psychological level [9,10], giving rise to them experiencing a low self-efficacy due to the limitations of the illness itself [8]. Furthermore, this population of MS sufferers is more sensitive to suffering cognitive dysfunctions and mental health problems, especially depression followed by anxiety, since this affects them at a larger scale than the general population (more than 20% of the population) [11,12]. Those people who suffered problems of mental health before the quarantine and social distancing may be more vulnerable to adverse psychological effects [1]. Recent research indicates that exposure risks are the cause of COVID-19 infection in the MS population. Younger people with a lower socioeconomic status will have a higher risk of exposure and are potential targets for educational intervention and medical support to limit exposure. Delays in therapies and the interruption of MS care have also been observed [13].

There is a lack of previous research that includes the consequences deriving from the COVID-19 virus pandemic at the physical and psychological level in people suffering from MS. This is why research into physical activity, resilience, coping and a sense of coherence has been selected, as these are the variables related to well-being and the promotion of health.

In people with MS, the degree of physical activity is usually reduced, making them less physically active than the general population [14]. It is related to the degree of general incapacity and the limitation for walking in people with MS [15]. In these patients, vigorous activities are reduced, leading to the predominance of light activities and the fact that they spend quite a lot of time being inactive during the day [16]. The reduced participation, the limitation of movement and the degree of incapacity factors mean that the physical activity of the MS population is less than that of the general population, and consequently they do not obtain the benefits that come from carrying out physical exercise, leading to a greater propensity for their suffering certain illnesses, especially cardiovascular ones [17]. It is necessary to promote physical activity and include it in intervention programs, since a greater degree of physical activity is related to personal factors such as sociodemographics, the level of education, their work situation, mechanisms for self-regulation and the perception of barriers, especially those related to physical activity [16].

Resilience is characterized by the ability to achieve, retain or recover a degree of physical or emotional health after adversity [17]. In people with MS, disability is associated with success in adapting and recuperation. Greater levels of resilience are associated with a reduction in fatigue (20% less), greater participation and exercise, greater social and financial support, a healthier diet and better psychological health [17]. People with MS have reported that higher levels of resilience are associated with healthy behaviors and a more successful adaptation to changes in the life of the patients that result from their illness [18]. This variable has been studied in different populations [19], but rarely in patients with MS.

The sense of coherence is understood as a way of perceiving the world that allows stressful factors to be faced [20]. MS is a chronic illness with an unpredictable evolution [21], which includes suffering physical limitations and psychosocial difficulties that affect the quality of life of these people, threatening their sense of cohesion [8,22]. A greater sense of cohesion gives rise to better mental health and a lower negative effect, as well as warding off depression [8,23].

People with MS use different coping strategies in the different stages of the illness. In the first stages, emotional and avoidance-type strategies predominate; meanwhile, in the intermediate stages of the illness, the use of more active coping strategies predominate [24]. On certain occasions, the use of emotional-type strategies are related to a better psychological adaptation and those of avoidance are related to the prevention of a depressive reaction through the rejection of negative thoughts about the future progression of the illness when they are used in the first stages following the diagnosis of the illness [23]. Psychological interventions centered on one’s resources to cope more effectively with the MS are necessary to bring a positive adaptation to the adverse situation [25].

Based on the aforementioned points, the following study has been carried out to obtain greater knowledge on the influence of this pandemic on the health/illness of people with MS. The objective will be to discover and analyze the relationships between physical activity, a sense of coherence, resilience and coping in people diagnosed with MS during the emergency health situation brought about by COVID-19.

## 2. Materials and Methods

A cross-sectional, descriptive and correlational study was conducted.

### 2.1. Participants

The population is made up of 1236 people with MS belonging to multiple sclerosis associations in Castilla y León (Spain) (See Table 1). Through a nonprobabilistic convenience sampling, 84 participants were selected.

### 2.2. Instruments Used to Collect Data

The short-format of the (IPAQ-SF) international questionnaire, validated in Spanish [26], is used with young adults and middle-aged people (15–69). It consists of four generic items to obtain information on physical activity related to health. It provides separate results for the three types of activity: vigorous intensive activities, activities of moderate intensity and walking. Obtaining the final result requires the totaling of the duration (in minutes) and frequency (in days) of these three types of activity, from which the physical activity is categorized in three levels: low, moderate and high. The “sitting time” is also registered. This is time dedicated to carrying out a sedentary activity [27].

The Resilience Scale, (ER-14), has 14 items to evaluate the degree of individual resilience [19]. The version was validated into Spanish by Sánchez–Teruel and Robles–Bello [28]. The ER-14 measures two factors: on the one hand, personal competence (11 items), and on the other hand, the acceptance of oneself and of life (three items). This scale presents a suitable internal consistency (α = 0.79) and validity of the calculated criterion with other measurements of general resilience (CD-RISC) (r = 0.87; *p* < 0.01). According to the scores: 98–82: very high resilience; 81–64: high resilience; 63–49: normal; 48–31: low; and between 30–14: very low. The reliability indices are suitable, demonstrating that it is a good instrument for measuring this variable [28].

The questionnaire on the Sense of Coherence (SOC 13) [20] is the Spanish-adapted version [29]. This scale consists of 13 items that are scored using a Likert-type scale of seven points. It contains three scales corresponding to the three dimensions of the construct: compression (α = 0.630), direction (α = 0.596) and significance (α = 0.594), and a total score (α = 0.799).

The questionnaire on coping COPE-28 [30] is the Spanish version (COPE-28) [31]. It is composed of 28 items scored using a Likert-type scale that goes from 0 (absolutely nothing) to 3 (a lot). These items are grouped together in pairs, producing 14 modes of coping: active coping (α = 0.452), instrumental support (α = 0.630), acceptance (α = 0.516), self-distraction (α = 0.482), venting (α = 0.302), planning (α = 0.585), religion (α = 0.880), denial (α = 0.458), self-blaming (α = 0.617), substance use, emotional support, positive reframing (α = 0.602), humor (α = 0.835) and behavioral disengagement (α = 0.654).

### 2.3. Procedure

The data for the study was gathered online by means of Google Forms (https://forms.gle/yu1gfoSX8h6L1HnV9). Prior to sending the questionnaire, all of the MS associations of Castilla y León (Spain) were contacted by telephone and e-mail. The members diagnosed with MS were requested to participate in the questionnaire using e-mail or WhatsApp. The gathering of the data took place between 28 March and 3 June 2020, during the period of confinement, due to the state of alarm imposed by the Spanish government for the management of the health crisis situation.

### 2.4. Data Analysis

The analysis of the data was descriptive, analytical and correlational. It was checked that the adjustment did not comply with the normal distribution of the data by means of the Kolmogorov–Smirnov test with the Lilliefors significance correction. For this reason, nonparametric tests were carried out (U of Mann–Whitney) for the comparisons of averages between the groups. Nonparametric correlations were carried out in order to evaluate the associations between the quantitative variables, and the Spearman rho coefficients were obtained. A value of *p* < 0.05 was considered statistically significant.

The database and the statistical analyses cited were carried out using the SPSS 26.0 (Statistical Package for the Social Sciences) computer program (IBM, New York, NY, USA).

### 2.5. Ethical Considerations

The people participating in the study were considered anonymous and the data confidential. Prior informed consent was requested for their collaboration. The data are collected and handled respecting all of their rights and guarantees in accordance with those established in the UE2016/679 Regulation and in the Constitutional Law 3/2018 of 5 December on the Protection of Personal Data and digital rights guarantees. The study was approved by the ethics committee (ETICA-ULE-016-2020) of the University of León (Spain), which guarantees that the questions are both ethical and legal.

## 3. Results

The sociodemographic characteristics of the participants are set out in Table 2. 84 people diagnosed with MS in the Spanish Autonomous Community of Castilla y León participated in the study. The results of the average and the standard deviation in terms of age were 46.9 ± 9.7 years old (M±SD). In relation to the clinical characteristics of the illness, the greatest part of the sample, 67.9%, was diagnosed with Relapsing Remittent Multiple Sclerosis (EMRR), followed by 25% diagnosed with its Secondary Progressive Multiple Sclerosis (EMPS). The descriptive values for the years of diagnosis of the MS were 13.9 ± 8.3 (average 15 years old, maximum 35 and minimum 1).

The results obtained for the physical activity variable are set out in Table 3. The U Mann–Whitney test has been carried out, and no differences have been found between men and women. As regards the scores for the questions on physical activity, 28 participants (33.3%) have scored high, 18 (21.4%) low and 29 (34.5%) have achieved a moderate degree of physical activity.

The descriptive statistics of the resilience, sense of coherence and coping variables are set out in Table 4. No differences have been found in the sex of the participants for the scores of these variables. In the case of resilience, 30 (35.7%) participants were perceived as having a very high resilience, 37 (44%) were evaluated as having high levels, 14 (16.7%) scored within what is considered normal, and 2 (2.4%) and 1 (1.2%) obtained low values or very low values, respectively.

Table 5 details the correlations between the variables of the study. The results indicate that physical activity does not correlate significantly with any of the other studied variables; however, although in some cases they are weak, correlations are observed between resilience, sense of coherence and coping. Thus, the highest levels of personal competence (CP), acceptance of oneself and of life (De) for resilience are related to the highest scores of compression (CO), direction (Dir) and significance (Sig) for the sense of coherence. As regards coping, the strategies for active coping (A), acceptance (Ac) and positive reframing (Pr) are positively related to the total and to the two dimensions of resilience of personal competence (CP) and acceptance of oneself and of life (AV), as well as to the factor of significance (Sig) of the sense of coherence variable. The strategies of denial (D), self-blaming (Sb) and behavioral disengagement (Bd) show negative correlations with resilience and the sense of coherence which have been evaluated in the study. Substance use as a strategy (Su) correlates negatively with resilience, in particular with the dimension of accepting oneself and life (De).

## 4. Discussion

The results revealed that the clinical and personal characteristics of the participants in the present study were similar to other studies conducted on patients with multiple sclerosis during the COVID-19 pandemic [1,13,32].

If we compare the time spent sitting down per week collected with the IPAQ of our sample with the study carried out by Moti et al. [33], which analyzed people with MS in America, we find that our population has a slightly higher value. Furthermore, our data show elevated levels of physical activity. These data seem to indicate a high participation in the activities proposed by the different associations and that they are concerned about their physical condition during this period of confinement. On the other hand, Hubbard et al. [34] observed that people with slight MS tended to do less physical activity in comparison with healthy people.

The results showed that people with multiple sclerosis who participate in associations presented high levels of resilience [19] overall and in the domains of personal competence and acceptance of oneself and of life. These values of adaptation to adverse situations are important for the well-being of people, as the studied sample went through 11 weeks of quarantine and time is considered as a predictive factor for acute stress disorder [3]. Furthermore, the effect of interventions that focus on promoting resilience and other personal resources are necessary to effectively cope with multiple sclerosis [25]. If the present study is compared to the work of Plowman et al. [17] in people with MS, the total resilience factors are very similar. On the contrary, the total resilience values and the I factor (personal competence) obtained in our sample are higher than those reached in the study carried out by Sánchez–Teruel et al. [28] in young healthy people. The case of populations with painful chronic pathologies such as fibromyalgia [35] highlights that our results are much higher (almost double) for this variable. It will be interesting to analyze the role of the associations in the promotion of the health of patients diagnosed with MS.

According to the data on the sense of coherence, those with multiple sclerosis who frequently attended associations in the Autonomous Community of Castilla y León had a total sense of coherence of 61.19 ± 12.252 during the confinement period. If we compare our data with the study of the sense of coherence in health personnel during the COVID-19 pandemic, we observe that our sample obtained higher mean values although they did not present symptoms of COVID [36]. Nonetheless, lower values than those of the present study are observed in populations with MS [37,38]. Conversely, persons with late effects of polio have obtained higher values, which could be due to their perception of the disability being less acute and to the fact that, upon having a later start, they have developed the capacity to understand, manage and be motivated when faced with stressful events and problems that come up during their lives [39]. If we compare our data with people without neurological problems and with a cardiac pathology, we observe that cardiac patients obtain worse values since they tend to be associated with other pathologies such as depression [40]. However, if we compare our sample with people older than 65–75, 76–80 and over 80, we see that all of the older people have higher scores in total (74 ± 3.44) and for decompression (27 ± 0.49), direction (23 ± 0.83) and significance (23 ± 1.09) [40].

The coping of the people with multiple sclerosis who participated in associations showed diverse levels according to the strategies used in comparison with people with multiple sclerosis who coped with the prognosis of their illness [29]; we observe that our population presented higher levels in terms of instrumental support 3.48 ± 1.52, substance use 3.52 ± 1.72, self-blaming 2.57 ± 2.16 and religion 4.55 ± 1.38. This leads us to think that they have less functional strategies when facing the COVID-19 situation due to their low self-esteem, perceived high levels of stress or psychological unease, as observed in French people facing serious illness or trauma [12]. In addition, people with MS can show increased discomfort due to having psychological problems due to alterations of cognitive dysfunctions, information processing, and immediate and episodic memory typical of MS [41]. Furthermore, the high values in religious coping lead us to think that they indicate a lower satisfaction with the decision that was taken to resolve the existence of stress [42]. Comparing our results on coping with values for healthy people, we find that emotional support, humor and positive revaluation present lower values in our study [43]. These data are logical because people with MS offer to undertake maladaptive coping strategies [12].

Finally, the results of this study indicate that the level of physical activity of a person with MS, which is measured in terms of the mastery of activity (vigorous, moderate), the total and the time spent sitting down, is not related to resilience, coping or sense of coherence in the social confinement situation that derives from the COVID-19 virus. However, we have found theoretical studies which support that psychological aspects can be influenced by physical activity [9,44].

Regarding the relationships between resilience and sense of coherence, this study observed that there was a positive relationship between them. This means that the most resilient people present greater levels of a sense of coherence and vice versa. This could benefit patients with MS, since there is evidence that supports a relationship between positive dimension factors (resilience and/or optimism) and subjective emotional well-being [45]. Recent research on the impact of COVID-19 on German public health supports our data, proposing to improve the sense of coherence to improve resilience to stressors [46]. According to the results of the relationships between coping and resilience, we observe that if a patient has a higher score in the strategies of active coping, acceptance, emotional support and planning, he or she will present greater levels of resilience and will have a greater capacity for adapting to adverse situations; that is, strategies for positive coping or task orientation are related to a better mental health [47,48]. In the same way, coping strategies such as denial, self-blame, substance abuse, venting and behavioral disconnection are negatively related to resilience, since they are strategies that maintain stress, as has been observed by other researchers [43,49]. Concerning the relationships between sense of coherence and coping, they depend on the strategies, as not everybody behaves in the same way. However, we can highlight the negative relationship between both venting and behavioral disengagement and the dimension of compression of the sense of coherence. According to recent studies, the behavior of our sample is the same as in Bedouin women older than 61 with little education and few possibilities for finding a job [50]. In both populations, one can observe that when there is a sensation of there being no control over the results or of being at the mercy of luck, it is probable that they will have a sensation of impotence and use passive or evasive strategies such as behavioral disconnection or the strategy of venting, which consists in verbally expressing the negative parts of a stressful situation. For all of the points mentioned above in relation to the COVID-19 scenario, researchers such as Ornell et al. support three main factors for developing mental health strategies: (1) multidisciplinary teams; (2) regular and precise clear communications about the outbreak situation; and (3) psychological counseling services through electronic applications for the community in general and especially for vulnerable people [4].

This study presents limitations, such as the method of sampling and the selection of the participants, which may exclude MS patients who do not belong to associations; furthermore, other variables such as the level of education, degree of incapacity and the medication used could have been included. Furthermore, it would be desirable to carry out longitudinal studies during similar adverse situations in people with multiple sclerosis, which might include variables such as resilience, sense of cohesion and coping strategies, as well as other more specific physical capacities altered by the illness, such as balance, walking or strength.

## 5. Conclusions

The majority of the participants in associations in Castilla y León (Spain) suffer from a type of Relapsing Remittent Multiple Sclerosis, followed by Secondary Progressive Multiple Sclerosis. In spite of the confinement/quarantine, the greatest part obtains high and moderate scores in physical activity. Although no relationship has been found between physical activity and the psychological variables, there is evidence of the influence of the latter on the well-being of the patients. It can be observed as those people who achieve high values in resilience, as well as in sense of coherence. Furthermore, the use of coping strategies in the sample confirms that active coping, acceptance, positive reframing, denial, self-blaming, behavioral disengagement and venting are negatively related to resilience and the sense of coherence.

This research may be useful in describing the degree of coping, sense of coherence, physical activity and coping strategies of people with multiple sclerosis in the confinement/quarantine situation deriving from the COVID-19 virus in Spain. Likewise, to detect possible problems and be able to establish psychological and or physical interventions for the associations of those affected with MS, this research might be repeated again in the future in order to promote health in similar situations.

## Figures and Tables

**Table 1:** Multiple sclerosis associations of the Castilla y León region (Spain).

PROVINCE	Associations name	WEB
Leon	SIL association for multiple sclerosis. Ponferrada ASILDEM.	https://aedem.org/quick-show/151-vacio/215-asociacie-em-de-fuentes-nuevas-le
	Leon multiple sclerosis association ALDEM.	https://esclerosismultipleleon.org/
Valladolid	Valladolid multiple sclerosis association AVEM.	https://www.emvalladolid.es/
Burgos	Association of relatives and sufferers of multiple sclerosis AFAEM.	https://www.esclerosismultipleburgos.org/contacto/
	Burgos multiple sclerosis association ASBEM.	http://www.asbemiranda.org/
	Ribera de Duero multiple sclerosis association AREM.	https://aedem.org/noticias-asociaciones/noticias-ribera-de-duero/76-asociaciibera-de-duero-de-esclerosis-mple-actividades-del-a003
Salamanca	Salamanca multiple sclerosis association ASDEM.	www.asdem.org/
Segovia	Segovia multiple sclerosis association ASGEM.	www.segoviaesclerosis.org/
Zamora	Zamora multiple sclerosis association AZDEM.	www.azdemzamora.es
Palencia	Palencia multiple sclerosis association APEM.	https://esclerosismultiple.com/entidades/asociacion-palentina-de-esclerosis-multiple-apem/
Avila	Avila multiple sclerosis association ADEMA.	www.ademavila.com/
Soria	Soria multiple sclerosis association ASOEM.	https://aedem.org/ini/1888-informe-bernat-soria

**Table 2:** Sample description.

		Total 84 (100%)
Gender	Man	34 (40.5%)
	Women	50 (59.5%)
City	Aranda de Duero	1 (1.2%)
	Ávila	4 (4.8%)
	Burgos	17 (20.2%)
	Miranda de Ebro	4 (4.8%)
	Palencia	13 (15.5%)
	Ponferrada	15 (17.9%)
	Segovia	4 (4.8%)
	Soria	6 (7.1%)
	Valladolid	20 (23.8%)
Type of multiple sclerosis	Primary Progressive	5 (6.0%)
	Progressive Recurrent	1 (1.2%)
	Secondary Progressive	21 (25.0%)
	Relapsing Remittent	57 (67.9%)
Number of people you live with	0	8 (9.5%)
	1	29 (34.5%)
	2	28 (33.3%)
	3	15 (17.9%)
	4	4 (4.8%)
Living with children under 14 years of age	0	63 (75.0%)
	1	13 (15.5%)
	2	7 (8.3%)
	3	1 (1.2%)
Have you had COVID-19?	Yes	0 (0%)
	No	68 (81.0%)
	Maybe	16 (19.0%)
Do you have COVID-19?	Yes	0 (0%)
	No	77 (91.7%)
	Maybe	7 (8.3%)
Have you lived or do you live with people with COVID-19?	Yes	0 (0%)
	No	71 (84.5%)
	Maybe	12 (14.3%)

**Table 3:** Descriptive statistics of physical activity in minutes per week.

Questionnaires		*n*	Minimum	Maximum	M ± SD	Me *	IR *
IPAQ *	Vigorous activity (V)	66	0	7200	1042.42 ± 1568.32	340	1480
	Moderate activity (M)	65	0	3600	870.77 ± 1005.62	540	1200
	Walk (W)	74	0	4258	956.0 ± 1158.65	495	1328.25
	TOTAL physical activity	75	0	10,890	2614.7 ± 2472.40	1986	3186
	Sitting time	61	15	1380	430.08 ± 282.59	360	270

Note: * minutes/week. M; Mean. SD; Standard Deviation. Me; Median. IR; Interquartile Ranges.

**Table 4:** Descriptive statistics of resilience, sense of coherence and coping.

Questionnaires		*n*	Items	Minimum	Maximum	M ± SD
**ER-14**	Personal competence	84	11	14	77	59.71 ± 11.26
	Acceptance of oneself and life	84	3	4	21	15.19 ± 3.90
	Total	84	24	18	97	74.90 ± 14.39
**SOC-13**	Compression	84	5	11	32	22.50 ± 5.13
	Direction	84	4	7	27	18.26 ± 4.79
	Signification	84	4	7	28	20.42 ± 4.31
	Total	84	13	29	82	61.18 ± 12.25
**COPE-28**	Active coping	84	2	0	6	4.37 ± 1.27
	Instrumental support	84	2	0	6	3.48 ± 1.52
	Acceptance	84	2	1	6	3.65 ± 1.59
	Self-distraction	84	2	0	6	2.80 ± 1.42
	Venting	84	2	0	6	1.20 ± 1.66
	Planning	84	2	0	6	3.54 ± 1.73
	Religion	84	2	0	6	4.55 ± 1.38
	Denial	84	2	0	6	0.90 ± 1.40
	Self-blaming	84	2	0	6	2.57 ± 2.16
	Substance use	84	2	0	6	3.52 ± 1.72
	Emotional support	84	2	0	6	1.63 ± 1.66
	Positive reframing	84	2	0	6	0.83 ± 1.19
	Humor	84	2	0	6	2.23 ± 1.52
	Behavioral disengagement	84	2	0	6	0.26 ± 0.97

Note: ER-14: Resilience Scale. SOC-13: Sense of Coherence. COPE-28: Coping. SD: standard deviation. M: mean.

**Table 5:** Spearman’s rho correlations of physical activity, resilience, sense of coherence and coping.

Questionnaires		IPAQ				ER-14			SOC-13			
		W	M	V	T	CP	DE	T	CO	Dir	Sig	T
**IPAQ**	W	1.000										
	M	0.406 **	1.000									
	V	0.074	0.447 **	1.000								
	T	0.601 **	0.815 **	0.656 **	1.000							
**ER-14**	CP	−0.003	0.055	0.112	0.113	1.000						
	De	−0.073	−0.014	0.066	0.006	0.724 **	1.000					
	T	−0.011	0.039	0.106	0.092	0.980 **	0.834 **	1.000				
**SOC-13**	Co	−0.001	−0.026	−0.088	−0.046	0.433 **	0.498 **	0.467 **	1.000			
	Dir	−0.103	−0.041	−0.011	−0.068	0.366 **	0.382 **	0.384 **	0.745 **	1.000		
	Sig	0.059	−0.062	0.112	0.009	0.426 **	0.390 **	0.445 **	0.517 **	0.500 **	1.000	
	T	−0.020	−0.036	−0.015	−0.043	0.467 **	0.489 **	0.496 **	0.891 **	0.877 **	0.772 **	1.000
**COPE-28**	A	0.041	0.038	−0.096	0.051	0.405 **	0.351 **	0.433 **	0.097	0.035	0.266 *	0.148
	I	−0.138	−0.183	0.061	−0.175	−0.018	−0.062	−0.030	−0.144	−0.130	−0.021	−0.129
	Ac	−0.104	0.094	−0.094	−0.015	0.587 **	0.564 **	0.620 **	0.359 **	0.223 *	0.373 **	0.376 **
	Sd	0.023	0.015	−0.001	0.062	0.127	−0.016	0.109	−0.105	−0.232 *	−0.310 **	−0.248 *
	V	−0.125	−0.229	−0.004	−0.111	0.023	−0.049	0.030	−0.234 *	−0.187	−0.124	−0.215 *
	P	−0.101	0.002	0.056	0.098	0.215 *	0.186	0.228 *	0.045	−0.015	0.131	0.070
	R	−0.014	0.011	0.020	−0.030	−0.038	−0.077	−0.044	−0.078	0.071	−0.007	−0.039
	D	0.058	0.063	0.043	0.009	−0.190	−0.248 *	−0.213	−0.332 **	−0.314 **	−0.312 **	−0.390 **
	Sb	−0.162	−0.072	0.003	−0.069	−0.115	−0.290 **	−0.155	−0.348 **	−0.317 **	−0.395 **	-0.428 **
	Su	−0.177	−0.113	0.175	−0.040	−0.088	−0.219 *	−0.127	−0.104	−0.113	−0.077	−0.124
	E	0.010	−0.018	0.117	0.052	−0.005	−0.042	−0.011	−0.087	−0.018	0.143	0.005
	Pr	0.064	0.070	0.236	0.197	0.518 **	0.352 **	0.517 **	0.136	0.095	0.278 *	0.166
	H	0.094	0.019	0.142	0.044	0.129	0.047	0.115	−0.051	−0.078	0.062	−0.021
	Bd	−0.133	0.014	0.061	−0.013	−0.394 **	−0.286 **	−0.392 **	−0.251 *	−0.220 *	−0.355 **	−0.323 **

Note; *: *p* < 0.05, **: *p* < 0.01, T: total, W: Walk, M: Moderate activity, V: Vigorous activity, CP: competence personal, De: Acceptance of oneself and of life, Co: Compression, Dir: Direction, Sig: Signification, A: active coping, I: instrumental support, Ac: acceptance, Sd: self-distraction, V: venting, P: planning, R: religion, D: denial, Sb: Self-blaming, Su: substance use, E: emotional support, Pr: Positive reframing, H: humor, Bd: behavioral disengagement.
